# Sorption of Total Petroleum Hydrocarbons in Microplastics

**DOI:** 10.3390/polym15092050

**Published:** 2023-04-26

**Authors:** Arely Areanely Cruz-Salas, Maribel Velasco-Pérez, Nayely Mendoza-Muñoz, Alethia Vázquez-Morillas, Margarita Beltrán-Villavicencio, Juan Carlos Alvarez-Zeferino, Sara Ojeda-Benítez

**Affiliations:** 1 Departamento de Energía, Universidad Autónoma Metropolitana, Unidad Azcapotzalco, Ciudad de México 02200, Mexico; 2 Laboratorio de Residuos Sólidos, Instituto de Ingeniería, Universidad Autónoma de Baja California, Campus Mexicali, Mexicali 21100, Mexico

**Keywords:** beach, Gulf of Mexico, kinetics, marine environments, organic pollutants

## Abstract

As is the case for many others in the world, Mexican seas face complex pollution challenges; two of the contaminants that require special attention for their prevalence, possible chemical interactions, and relation to the country’s economy are leaked petroleum and microplastics (MP). This research assessed the sorption of total petroleum hydrocarbons (TPH) as fuel oil on microplastics in laboratory and field scenarios. Preliminary tests allowed the development and validation of a methodology to measure the sorbed fuel oil by Soxhlet extraction, with a 99.65% recovery rate. The amount of TPH sorbed in the lab followed the order LDPE > PS > PP > PVC > PET > HDPE, with the highest concentration found on LDPE. The sorption of fuel oil on microplastics is correlated to the surface area of the plastic particles and could also be related to the crystallinity of plastics. Sorption, for all plastics, was consistent with a second-order kinetic model. The analysis of field samples collected on beaches of the Gulf of Mexico varied from 1660 to 35,258 mg/kg MP. It must be noticed that, unlike others, this research quantified a family of contaminants, which could explain the high concentrations observed on microplastics.

## 1. Introduction

Plastics are valuable materials for industrial, commercial, medical, and technological applications. In 2020, the world production reached 367 million tons [1]. The increasing production of plastics causes a corresponding growth in plastic waste. Europe reported a post-consumer plastic waste production of 29.5 million tons, of which 42% was used for energy recovery, 34.6% was recycled, and 23.4% was landfilled [1].

In 2019 in Mexico, the plastic industry contributed 0.4% to the gross domestic product, placing this country as the 12th producer and the 11th consumer of plastics globally [2]. In 2020, 43.8 million tons of municipal solid waste (MSW) were generated in Mexico, with 12.9% (18.6 million tons) of plastic waste [3]. This country does not produce official statistics for plastic waste treatment, including recycling and final disposal. However, considering that 71.9% of MSW was taken to final disposal sites (FDS) and that 47.8% of the FDS do not have the basic infrastructure to prevent pollution [3], contamination potential from waste management in Mexico is relevant.

Plastic waste can reach the environment due to mismanagement, transport by waterways, and natural phenomena. Plastics can enter the ocean through wind, inland waterways (rivers, lakes, or channels), or tides [4]. An environmental risk associated with plastics’ presence in natural ecosystems is the formation of microplastics (MP), particles < 5 mm that can be produced in this size or formed by the fragmentation of discarded plastic waste. MPs vary in size, shape, color, density, and chemical composition [5,6]. Once in the ocean, MPs can be transported by marine currents [4], float, stay in the water column, or settle in beaches and marine sediments due to their density, which can be modified by biofilm formation [7]. Marine organisms can ingest MP because of their size. MPs sorb and accumulate persistent organic pollutants (POP) and other hydrophobic compounds in the surrounding environment [8,9]. Due to this, sometimes, MPs can be considered as a bioremediation strategy to eliminate contaminants present in the aqueous medium, in the same way composite materials are used to remove organic pollutants from aqueous media. For instance, cotton fiber covered with chitosan [10], the functional organic ligand of 4-tert-Octyl-4-((phenyl)diazenyl)phenol (TPDP) with mesoporous silica [11] and the chemical ligand of N,N–bis(salicylidene)1,2–bis(2–aminophenylthio)ethane (BSBAE) also with mesoporous silica [12] have shown high removal of Remazol Brilliant Red F3B (RR), copper (II) and cadmium (II), respectively. However, if the biota ingests MP with pollutants, they can introduce toxic compounds into the trophic chain, leading to chemical, ecological, and biological alterations in species and ecosystems [5,13], leading to bioaccumulation in organisms [14]. Examples of the adverse effects of MP include mechanical injuries and alteration in enzymatic biomarkers in *Scrobicularia plana* exposed to benzo(a)pyrene (BaP) and perfluorooctanesulfonic acid (PFOS) sorbed in low-density polyethylene (LDPE) [15]; decreases in embryo survival and growth, as well as behavior disorders for the fish *Oryzias melastigma* after exposition to PFOS, BaP, and benzophenone sorbed in polyethylene [16]. Although it has been proposed that this phenomenon occurs mainly in coastal waters [9], pollutants sorbed on MP can be transported to pristine ecosystems, where they can desorb. MP could increase the residence time of pollutants in organisms, contributing to the accumulation and chronic exposure of macro and microorganisms [17]. Due to the high surface/volume ratio of MP, concentrations of hydrophobic pollutants, such as polychlorinated biphenyls (PCB), dichlorodiphenyltrichloroethane (DDT), and polycyclic aromatic hydrocarbons (PAH), can be higher than those found on larger plastics in the surrounding water [18].

The presence of PAHs on MPs found in natural environments is well documented. Concentrations of 640 ng PAH/g MP and 1200–6200 ng PAH/g MP have been reported in albatrosses on Guadalupe Island in Mexico and San Gabriel River in California, United States [19]. In the Canary Islands, Spain, pellets presented 52.1–17,023.6 ng PAH/g MP, and plastic fragments presented 35.1–8725.8 ng PAH/g MP [9]. Analysis of MP collected on the Portuguese beaches of Cresmina and Thela showed 91.3–285.1 ng BaP/g MP [8]. On the MP found in surface waters in the Bohai and Huanghai seas, the concentration of PAH was 3400–119,000 ng PAH/g MP [20]; the primary source of pollutants was attributed to oil and gasoline. These data show that PAHs are found on MP in different types of ecosystems with a wide range of concentrations.

On the other hand, pollution by leaks, spills, and mismanagement of hydrocarbons during extraction and production is a relevant environmental problem in Mexico. There have been 3350 hydrocarbon pollution events from 2016 to 2020, with 931 in 2020 alone. In 2020, 77.4% of hydrocarbon pollution occurred during exploration and production [21].

Mixtures of hydrocarbons such as crude oil and some of its fractions are usually described and quantified as total petrol hydrocarbons (TPH). This group of compounds enters organisms through ingestion, inhalation, or dermal absorption. Due to their low water solubility and high lipid solubility, TPHs accumulate in organisms and organic matter in particles and sediments, becoming more susceptible to bioaccumulation [22]. TPHs include compounds such as PAH and diesel [20]. PAHs are formed during the incomplete combustion of organic compounds such as fossil fuels; these chemicals have carcinogenic and mutagenic effects in humans [23]. The individual effect of 18 aromatic hydrocarbons was studied on *Vibrio fischeri*, a bioluminescence bacteria found in symbiosis with marine animals such as mollusks [24]. It was found that the baseline toxicity model well explains luminescence inhibition by petroleum hydrocarbons. Luminescence inhibition of 50% was observed for individual petroleum hydrocarbons with chemical activities between 0.01 and 0.1. However, since baseline toxicity is additive in concentration, the toxicity cutoff observed for individual compounds may not be valid for mixtures.

This research aimed to evaluate the sorption of fuel oil on MP in a laboratory-scale simulated marine environment and to investigate sorption kinetics and the concentration of organic compounds on MP from beaches in the Gulf of Mexico (a region with a vast petroleum industry).

## 2. Materials and Methods

The methodology consisted of the following six steps: (1) the preparation of virgin microplastics (VMP), (2) determination of the concentration of organic compounds, (3) measurement of the surface area of VMP, (4) sorption of fuel oil on VMP in laboratory conditions, (5) determination of the sorption kinetics of fuel oil on VMP, and (6) determination of the concentration of organic compounds on MP collected on Mexican beaches (real microplastics, RMP). For the determination of the TPH concentration, a technique frequently used in many laboratories worldwide was used. This technique has the following advantages: it is easy to perform, does not require expensive equipment or chemical substances, and only requires equipment found in most laboratories (such as flasks, heating and stirring racks, stirrers, and universal supports, to name a few), and the analysis time is relatively short.

### 2.1. Preparation of Virgin Microplastics

**Figure 1:**
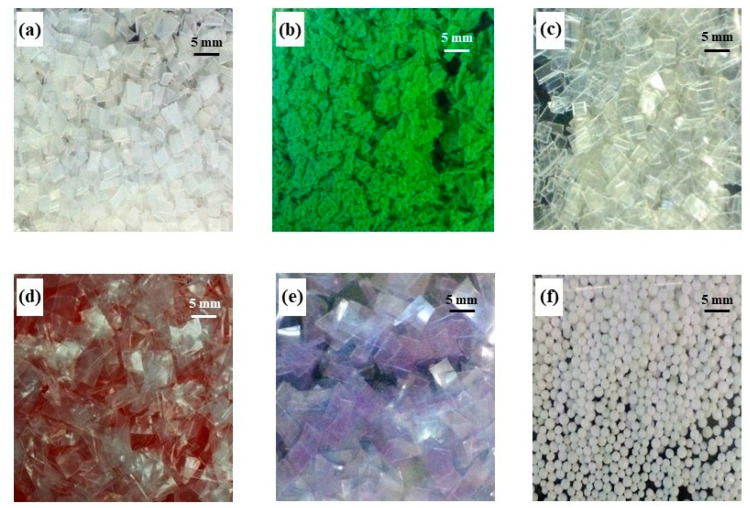
Types of virgin microplastics used in this research. (**a**) HDPE, (**b**) PP, (**c**) PET, (**d**) LDPE, (**e**) PVC, and (**f**) PS.

Most studies on the MP sorption of pollutants from aqueous medium use reagent-grade microplastics [25]. However, this study uses MP from virgin post-consumer plastic. VMPs were cut and then sieved in a size mesh of around 4.75 mm. High-density polyethylene (HDPE) was obtained from yogurt containers, polypropylene (PP) from carrier bags from Walmart^®^, polyethylene terephthalate (PET) from disposable water bottles, low-density polyethylene (LDPE) from clear carrier bags, polyvinyl chloride (PVC) from crystal transparent film, and polystyrene (PS) from Styrofoam balls (Figure 1).

### 2.2. Method for the Determination of the Concentration of Organic Compounds

#### 2.2.1. Determination of the Concentration of Fuel Oil in the Liquid Phase

The concentration of fuel oil, measured as the total organic carbon (TOC), in the liquid phase was determined by a TOC Analyzer with a Tekmar Dohrmann-Apollo 9000 model DRB-200 [26]. The operation conditions of the TOC analyzer were as follows: temperature combustion of 368.15 K and analysis time of 12 min. The volatility of the compounds was accounted for by determining the concentration of TOC in aerated and unaerated reactors.

#### 2.2.2. Determination of the Concentration of Fuel Oil on Microplastics

The concentration of organic compounds (OC) on microplastics was determined with Soxhlet extraction [27]. In Soxhlet extraction, the solid matrix (MP in this case) is placed in a thimble, a solvent is heated under reflux, solvent vapors rise through the thimble, and then the solvent cools and condensates fall in the thimble.

This method has been used to measure PAH and TPH in soil samples [28], but also to quantify concentrations of various OC (PAH, PCB, pesticides, aliphatic hydrocarbons, dichlorodiphenyldichloroethylene, among others) on MP recovered from beaches [19,29]. Since a study found no difference in the concentration of hydrocarbons and petroleum fractions using extraction times of 4, 8, 12, and 16 h [30], the time selected for the Soxhlet extraction was 4 h. Extraction was performed with 1 g of MP, at 318.15 K and with 120 mL of solvent (dichloromethane).

The concentration of fuel oil on VPM and RMP was calculated with Equation (1),

(1) $$q_{e}=\frac{V\;\left(\;Co-Ce\;\right)}{\;M}\;\;\;$$

where *q_e_* is the sorption capacity at equilibrium (mg/kg), *V* is the volume of the liquid phase (L), *C*_0_ is the initial liquid phase concentration of fuel oil at the initial time, *C_e_* is the liquid phase concentration of fuel oil at equilibrium and M is the mass of MP (kg). *C_e_* and *C*_0_ were determined as described in Section 2.2.1.

It was verified with Equation (2) that the initial mass of fuel oil (*m_initial_*) was equal to the sum of the mass of fuel oil sorbed on MP (*m_MP_*) and the mass of fuel oil remaining in the liquid phase (*m_lp_*).

(2) $$\%\;Recovery=\frac{m_{lp}+m_{MP}}{m_{initial}}\times 100\;$$

### 2.3. Determination of Surface Area, Average Pore Diameter, and Pore Volume of Virgin Microplastics

Surface area, average pore diameter, and pore volume of VMP were determined with physisorption analysis (N_2_ and 77 K). The surface area, diameter, and volume of pores were calculated by observing the adsorption of a gas (N_2_, for instance) on the particles of the solid (the sorbent) [31]. A Belsorp Max analyzer by MicrotracBEL Corp (Osaka, Japan) was used for physisorption on VMP; the surface area was calculated with the Brunauer–Emmett–Teller (BET) method, and pore size distribution with the Barrett–Joyner–Halenda (BJH) method. Preparation of samples consisted of drying VMP at 378.15 K for 24 h.

### 2.4. Evaluation of Sorption of Fuel Oil on Virgin Microplastics in Laboratory Conditions

The following procedure was undertaken for a fuel oil and synthetic seawater mixture without supernatant. Fuel oil (between 10 and 30 mL) was added to 500 mL of deionized water; then, this mixture was shaken for 2 h; after this time, the supernatant of the mixture was removed with a separation funnel. The remaining mixture was used to prepare emulsions with different concentrations of THP. The concentration of TPH (measured as TOC) of fuel oil and synthetic seawater emulsions was determined as described in Section 2.2.1.

Synthetic seawater was prepared with a method by Cifuentes et al. [32] and had the following chemical composition: 24 g/L sodium chloride (NaCl), 5 g/L magnesium chloride (MgCl_2_), 4 g/L sodium sulfate (Na_2_SO_4_), 1.1 g/L calcium chloride (CaCl_2_), 0.7 g/L potassium chloride (KCl), 0.2 g/L sodium bicarbonate (NaHCO_3_), 0.096 g/L sodium bromide (NaBr), 0.026 g/L boric acid (H_3_BO_3_), 0.024 g/L strontium chloride (SrCl_2_), and 0.003 g/L sodium fluoride (NaF) [32].

VMPs were placed in 500 mL glass reactors, and 150 mL of the fuel oil and synthetic seawater emulsion was added. Different reactors were used for each type of VMPs (HDPE, PP, PET, LDPE, PVC, and PS) and each initial concentration of fuel oil. VMPs were added at a 2% weight concentration. Each reactor was aerated for 8 h per day for seven days to simulate oxygenation conditions in real seawater; the air was added with the glass tubing. This experiment was performed in triplicate. No separation of the mixture of fuel oil and synthetic seawater was observed during the sorption experiments.

### 2.5. Sorption Kinetics of Fuel Oil on Microplastics

Sorption kinetics experiments were carried out with three types of plastic and one fuel oil concentration. The types of plastic selected for the kinetic study were the three with the highest sorption capacity in the sorption experiment conducted in Section 2.4. The fuel oil concentration used was the concentration that presented the highest sorption in the experiments conducted in Section 2.4. Each type of VMP was placed in a glass reactor with a mixture of fuel oil and synthetic seawater for 96 h. Samples of 25 mL of the emulsion were taken at 3, 6, 12, 24, 48, 72, and 96 h. This experiment was performed in triplicate. Experimental data were fitted to the pseudo first-, pseudo second-order, and intraparticle diffusion (ID) kinetic models (Table 1).

### 2.6. Determination of the Concentration of Organic Compounds in Real Microplastics

#### 2.6.1. Sampling of Microplastics

**Figure 2:**
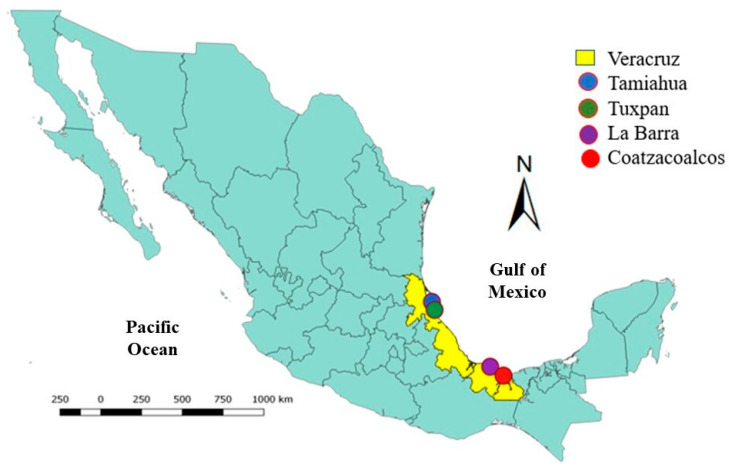
Location of the beaches where microplastics were sampled.

RMPs were sampled from the following beaches located in the Mexican Gulf: Tamiahua (21°17′23.4″ N, 97°25′12.06″ O), Tuxpan (20°58′28.5″ N, 97°18′24.84″ O), La Barra de Sontecomapan (18°33′45″ N, 95°0′28.74″ O), and Coatzacoalcos (18°9′7.3″ N, 94°27′7.75″ O) (Figure 2). Samples were collected in April 2018 at 6 a.m.

Sampling of RMPs was carried out with the following procedure. First, the beach’s high tide line was identified in an area polluted with OC. Then, a rope with a longitude of 100 m was placed parallel to the high tide line. Next, RMPs were collected with metallic tweezers along the 100 m transect. The collected RMPs were stored in glass containers with cork stoppers. This process was repeated in a non-polluted region of the beach to collect RMPs free of OC (to be used as blanks).

#### 2.6.2. Extraction of Fuel Oil from Microplastics

The sand was removed from the RMPs with deionized water. Then, RMPs were dried in an oven at 333.15 K for 2 h. Next, Soxhlet extraction was carried out [27]. Dichloromethane was used as a solvent to extract OC from the RMP (120 mL of solvent per 1 g of MP), and extraction was carried out for 4 h after the first cycle.

Determination of the concentration of OC in the RMP was estimated with Equation (1). The concentration of OC in blanks (RMP collected in the non-polluted area of beaches) was deducted from the concentration of OC from the polluted areas of the beaches. This part was carried out to account for biofilm and other organic matter on the MP.

## 3. Results and Discussion

### 3.1. Validation of the Method Used for the Determination of the Concentration of Organic Compounds in Emulsions

The fuel oil saturation concentration in water was determined by emulsifying different amounts of fuel oil in 500 mL of deionized water. It was concluded that the maximum concentration of fuel oil that could be emulsified in water was 150 ± 5.98 mg/L. Hence, the highest concentration studied in this research was around that value. In addition, the percentage of recovery for fuel oil was determined in different types of plastics to validate the analytical methods used in this research. The experiment was conducted with six types of plastics (PS, HDPE, PET, PP, LDPE, PVC) and four concentrations of fuel oil (126.35 ± 10.12 mg/L, 14.38 ± 2.20 mg/L, 1.93 ± 0.30 mg/L, 0.23 ± 0.10 mg/L) in triplicate. Since the recovery rate ranged from 87.03 to 99.88%, it was concluded that the analytical methods were robust.

### 3.2. Sorption of Fuel Oil on Virgin Microplastics in Laboratory Conditions

Table 2 presents the concentration (*q_e_*) of fuel oil (average ± standard deviation) on VMP after contact with synthetic seawater with different initial concentrations of fuel oil. The mixture of fuel oil and synthetic seawater remained emulsified during the experiment. This and the continuous mixing of the emulsion due to aeration allowed a similar interaction between fuel oil and different types of plastics, independently of the density of plastics. In natural environments, the density of plastics affects their position in the water column, plastics with a smaller density than seawater float, and plastics with a higher density sink. Biofilm formation on the MP’s surface affects the MP’s density and its interaction with the chemical substances present in seawater. This research did not measure or control the droplet size of fuel oil emulsified in synthetic seawater.

PS, PP, and LDPE sorbed the highest amounts of fuel oil. At the highest initial fuel oil concentration evaluated (126.35 ± 10.12 mg/L), the highest sorption was obtained with LDPE (2914.39 ± 359.49 mg/kg), while the lowest was with HDPE (1744.11 ± 119.36). In addition, at the lowest initial fuel oil concentration (0.23 ± 0.10 mg/L), the lowest sorption was obtained with PET (50.78 ± 4.13 mg/kg). VMP presented a significant change in color after exposition to fuel oil.

The toxicity of TPH depends not only on the concentration of petroleum hydrocarbons, but also on the compounds present in the mix [35]. The composition of TPH mixtures varies with sources of petroleum hydrocarbons or changes with time due to weathering and transport phenomena, among other factors. Hence, mixtures with the same TPH concentration may have different risks to the environment and human beings.

TPH are non-polar, hydrophobic, and lipophilic compounds with low solubility. TPH solubility decreases with the number of rings. Solubility influences the migration of pollutants in soil, superficial water, and groundwater. It has been demonstrated that PS and PE microplastics sorb low molecular weight PAHs with two or three rings [20].

It has been proposed that PS and PE microplastics can sorb high quantities of PAHs and other organic compounds [36,37]. In 2018, PAH concentrations between 6.9 and 77 mg /kg MP in PS and PE pellets were reported in China [20]. Other studies have reported the high sorption of various compounds on PS and PE. A study in fresh and seawater found that MP sorbed hydrophobic organic compounds [38]. The sorption capacity of MP increased with aging and salinity and decreased with size; sorption was between 10 and 100 times higher in nanoplastics than in microplastics (due to the surface area to volume ratio) [38].

The concentrations of organic compounds sorbed on MP reported in the literature are significantly lower than those obtained in this study. This may be explained by the relatively high concentration of emulsified fuel oil used in this study (126.35 ± 10.12 mg/L). Weathering of MP could also explain the differences in the values reported in the literature; in natural environments, MPs are subjected to weathering, which modifies their affinity for hydrophobic pollutants due to the introduction of oxygen-containing groups [39]. Finally, the density of MPs can affect their position in the water column. In this research, fuel oil sorption experiments were carried out with aeration. The mix of fuel oil, synthetic seawater, and MP was constantly agitated by air. Aeration did not allow the MPs with a low density to float or the MPs with a high density to sink. Other factors that affect MP’s position in the water column are biofilm formation on MP and marine currents. In natural environments, these factors affect the interaction of chemical substances (as pollutants) and MP.

### 3.3. Surface Area of Virgin Microplastics and Sorption of Fuel Oil

The surface area, average pore diameter, and pore volume of VMP are presented in Table 3; these properties were used for the kinetics study. Since the pore size for all the VMPs evaluated was between 2 and 50 nm, the materials were classified as mesoporous [31]. On the other hand, the sorption data presented in Table 2 indicate that for an initial concentration of fuel oil of 126.35 ± 10.12 mg/L, sorption decreased in the following order: LDPE (2914.39 ± 359.49 mg/kg) > PS (2894.48 ± 484.76 mg/kg) > PVC (2543.01 ± 422.39 mg/kg) > PP (2255.91 ± 264.34 mg/kg) > PET (1844.93 ± 502.15 mg/kg) > HDPE (1744.11 ± 119.36 mg/kg). Other initial concentrations of fuel oil presented a similar trend (Table 2). MP from LDPE (from 64.89 ± 0.40 mg/kg to 2914.39 ± 359.49 mg/kg), PS (from 62.80 ± 0.08 mg/kg to 2894.48 ± 484.76 mg/kg) and PP (from 63.38 ± 1.23 mg/kg to 2255.91 ± 264.34 mg/kg) presented the highest sorption capacities in the range of the fuel oil concentrations studied; these VMPs also presented the highest surface areas (PS: 17.45 m^2^/g, LDPE: 13.25 m^2^/g, PP: 11.23 m^2^/g). Several studies have found that surface area affects the sorption of organic compounds on MP. Furthermore, MP properties could influence sorption behavior.

In the following paragraphs, the effect of surface area on the sorption of organic compounds on MP is presented. Goedecke et al. found that the sorption of metformin, a type-2 diabetes drug, increased when PP was cryo-milled (particle size: 0.2–0.6 mm) [40,41]. Furthermore, a study of 3,6-dibromocarbazole and 1,3,6,8-tetrabromocarbazole sorption on MP found the highest sorption capacity at a small particle size (0.45–5 mm) [42]. Wang and Wang studied pyrene sorption on MP, with sorption decreasing in the order PE > PS > PVC [33]. This behavior was attributed to the physicochemical properties of plastics. For instance, PE presented the highest surface area (6.91 m^2^/g), followed by PS (2.35 m^2^/g), and PVC (1.87 m^2^/g). In our study, the highest sorption of fuel oil at an initial concentration of 126.35 ± 10.12 mg/L was obtained for LDPE, PS, and PVC, in agreement with previous reports [33].

The porosity of materials could also affect the sorption of pollutants. Materials have pores of different sizes, and organic pollutants may enter those pores and be trapped [39]. It has been found that oxytetracycline sorption in weathered PS microplastics was enhanced by the high specific and micropore area and the degree of oxidation of MP [43]. The sorption of 19 different compounds (pesticides, pharmaceuticals, and personal care products) on PE and PS microplastics was studied, finding that the high porosity of PS enhanced its sorption capacity [44]. Other researchers found that the high sorption capacity of nitrobenzene and naphthalene on PP microplastics was due to their porosity and high surface area (3.327 m^2^/g) [45].

The type of polymer may also influence the sorption of some pollutants on MP [46,47]; it was found that non-polar compounds (perfluoro octane sulfonate and perfluoro octane sulfonamide) present higher affinity for PE (polar) than for PVC (nonpolar) [48]. In several studies, PE and PS have presented high values of sorption for pollutants [20,36,38,45].

Hüffer and Hofmann investigated the sorption of seven aliphatic and aromatic organic probe sorbates on four types of MP. Sorption increased in the order polyamide < PE < PVC < PS, suggesting hydrophobic interactions are highly important [49]. When changes in energy due to interactions between compound plastic are more favorable than for interactions between compound aqueous phases, the highest concentration of the compound at equilibrium will be on the plastic [40].

In conclusion, the order and rate of sorption depend on the hydrophobicity of compounds, type of plastic, aging of plastics (virgin materials or aged), and properties of the aqueous phase (temperature, pH, salinity, and composition) [50]. Wang et al. published a detailed discussion on the factors affecting the sorption of organic pollutants on MP [39].

### 3.4. Sorption Kinetics of Fuel Oil on Virgin Microplastics

**Figure 3:**
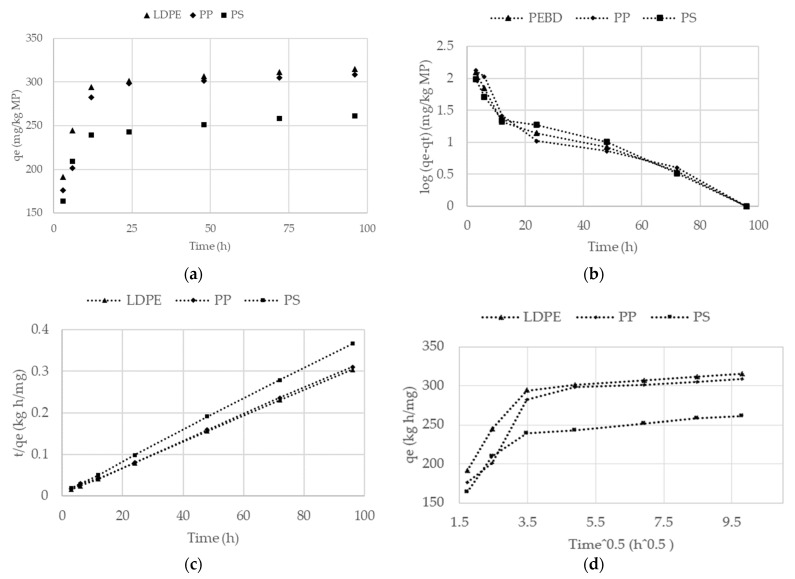
Sorption kinetics. (**a**) Experimental data for sorption of fuel oil on virgin microplastics, (**b**) pseudo first-order kinetic model, (**c**) pseudo second-order kinetic model, and (**d**) intraparticle diffusion model.

Figure 3 shows experimental data for sorption experiments and experimental data fitted to pseudo first-, pseudo second-order kinetic models and intraparticle diffusion. Table 4 presents the sorption parameters for the kinetic models evaluated in this research. Kinetics were studied only for fuel oil sorption on PS, PP, and LDPE VPM because this type of plastic presented the highest sorption in previous experiments.

The sorption capacity (*q_e_*) of TPH on VMP followed the order LDPE (315.52 mg/kg) > PP (308.73 mg/kg) > PS (315.52 mg/kg); this can be observed in Figure 3a. The kinetic model that presents the lowest linear correlation, between VMP and fuel oil, was that of intraparticle diffusion (ID). In this model, the principle is that if the straight line passes through the origin, intraparticle diffusion is a rate-limiting step that controls the adsorption process, whereas if the line does not pass through the origin, both intraparticle diffusion and external diffusion play an important role in sorption [34]. Factors such as sorbent particle size, temperature, solute concentration, to mention a few also have an effect on sorption [34]. The VMP evaluated presented a very high linear correlation with the pseudo second-order kinetic model, suggesting that sorption may involve electron sharing or transfer between the sorbent and the sorbate [42]. The very high correlation with the pseudo second-order model indicates that the sorption of fuel oil on MP depends on sorption capacity and that the rate-limiting step is chemisorption. Several studies have found similar results [51,52]. For instance, these include fipronil on non-degradable and biodegradable MP (R^2^ = 0.953–0.998) [53]; pyrene on PE, PS, and PVC MP (R^2^ > 0.99) [33]; phenanthrene on PE and nylon fibers from a mariculture farm in Xiangshan Bay, China (R^2^ > 0.99); and fipronil on PBS, polylactic acid (PLA), PP, PE, PS, and PVC (R^2^ = 0.953–0.998) [53]. However, the results of the kinetic models may have been influenced by fuel oil uptake on the MP surface due to the high oleophobicity of MP.

The sorption capacity of organic compounds on MP has been recently studied through partitioning [33,48,54]. The octanol–water coefficient has been effectively used to study hydrophobic partitioning interactions between organic pollutants and MP.

Regarding contact time, in this research, 93–97% of sorption occurred mainly during the first 24 h. After this period, the sorption rate decreased significantly. In the literature, equilibrium sorption times vary widely. Bakir et al. studied the sorption of phenanthrene and DDT on PVC and PE (particle size 200–250 μm); these compounds reached equilibrium in 24 h, except for the sorption of DDT on PE that required 48 h [55]. Wang et al. found that the phenanthrene sorption on nylon MP achieved equilibrium at 24 h [40]. Guo et al. studied the sorption of tylosin on PE, PP, PS, and PVC, with an equilibrium time of 36 h [56]. The sorption of pyrene on PE, PS, and PVC reached equilibrium in 48 h [33]. Zhao et al. found an equilibrium sorption time of 5 days for PAH on polar (PBS, PCL; and PU) and non-polar (PS) microplastics [57].

### 3.5. Sampling and Sorption Evaluation of Organic Compound on Real Microplastics

Table 5 presents the concentration of organic compounds on RMP sampled at four beaches in Veracruz, Mexico. The concentration reported here was calculated by subtracting the concentration of organic compounds in the blanks from the concentration in the samples (to account for the contribution of biofilm in the samples).

The concentration of organic compounds on RMP ranged from 1660.85 to 32,258.81 mg/kg. In the literature, the concentration of organic pollutants varies widely, especially in samples from natural environments. Variation in the concentration of organic compounds in RMP could be due to the type of organic compound studied, the type of MP studied, the chemistry of the medium in which sorption takes place, weathering of samples of MP, and the method used to extract the compounds from MP. In a study where MP samples were cleaned with a 30% hydrogen peroxide (H_2_O_2_) solution before extraction, concentrations of PAH from 3.4 to 119 mg/kg were found [20]. Bouhroum et al. reported average PAH concentrations of 5.8 × 10^−2^ mg/kg on PE and 0.142 mg/kg on PET in samples collected in the North Atlantic gyre. The extraction of PAH was carried out with a mixture of solvents (dichloromethane and heptane) [58]. Furthermore, concentrations of organic compounds of 1–10,000 mg/kg in microplastics from the Pacific Ocean have been reported before, with extraction with dichloromethane [59].

## 4. Conclusions

The interactions between microplastics and other pollutants found in the marine environment are a very relevant topic due to the potential of plastic particles to act as carriers of contaminants in the food chain. Different researchers have suggested that specific organic and inorganic compounds can be sorbed on the surface of plastics.

In this study, the approach was to analyze a family of contaminants found in the Gulf of Mexico due to leaks occurring during petroleum extraction and processing. Analyzing samples artificially polluted with fuel oil in the lab allowed us to define a methodology that efficiently recovered the studied TPH fraction. This extraction technique could be used with other organic pollutants, especially when they are quantified together.

The interactions between plastics and organic pollutants are complex because different factors can influence the sorption process, such as the hydrophobicity of the compounds, the composition and physical characteristics of the plastics, the polarity of the species involved, and the properties of the aqueous phase. On the other hand, the possible sorption–desorption process and kinetics could significantly influence the ecotoxicity associated with the ingestion of microplastics. The presence in the marine environment of different pollutants could simultaneously result in competition or synergistic effects; more research is needed to understand these dynamics. Measuring families of compounds together can be a useful tool to achieve this.

The high concentration of OC found in the field samples on beaches polluted by hydrocarbons proves the high level of interaction between the studied contaminants. These findings are relevant, as both are common on the beaches of the Gulf of Mexico, where the petrochemical industry has a high presence. Further research could help us to better understand the magnitude of the problem and its potential effects on natural ecosystems.

## Figures and Tables

**Table 1:** Sorption kinetics models used in this research [33,34].

Model	Equation	Linear Equation
Pseudo first-order model	$\frac{dq}{dt}=k_{1}\left(q_{e}-q_{t}\right)$	$\log\left(q_{e}-q_{t}\right)=\log\left(q_{e}\right)-\frac{k_{1}}{2.3}t$
Pseudo second-order model	$\frac{dq}{dt}=k_{2}{\left(q_{e}-q_{t}\right)}^{2}$	$\frac{t}{q_{t}}=\frac{1}{k_{2}q_{e}{}^{2}}+\frac{1}{q_{e}}t$
Intraparticle diffusion (ID)	-	$q_{t}=k_{3}t^{0.5}+C$

*k*_1_ = rate constant of the pseudo first-order model (1/h), *k*_2_ = rate constant of the pseudo second-order model (kg/mg h), *k*_3_ = rate constant of ID model (mg/kg h^0.5^), *t* = time (h), *C* = constant of the ID model (mg/kg), and *q_e_* = sorption of fuel oil at equilibrium in the solid phase (mg/kg).

**Table 2:** Concentration of fuel oil (measured as organic carbon in mg/kg) sorbed on different types of virgin microplastics.

Plastic Type	Initial Concentration of Fuel Oil in Emulsions			
	126.35 ± 10.12 mg/L	14.38 ± 2.20 mg/L	1.93 ± 0.39 mg/L	0.23 ± 0.10 mg/L
PS	2894.48 ± 484.76	1935.44 ± 2.28	224.55 ± 3.9	62.80 ± 0.08
HDPE	1744.11 ± 119.36	1970.01 ± 13.15	207.90 ± 25.43	59.08 ± 1.97
PET	1844.93 ± 502.15	1892.01 ± 19.0	186.13 ± 9.05	50.78 ± 4.13
PP	2255.91 ± 264.34	1964.56 ± 17.55	239.52 ± 21.74	63.38 ± 1.23
LDPE	2914.39 ± 359.49	2014.64 ± 34.67	223.71 ± 1.29	64.89 ± 0.40
PVC	2543.01 ± 422.39	1704.27 ± 3.22	198.57 ± 13.11	55.90 ± 3.24

**Table 3:** Surface area and pore size and volume of virgin microplastics.

Plastic Type	Surface Area (m^2^/g)	Average Pore Diameter (nm)	Pore Volume (10^−2^ cm^3^/g)
PS	17.45	5.57	1.42
HDPE	2.85	11.66	2.43
PET	6.55	4.91	0.83
PP	11.23	5.05	0.96
LDPE	13.25	4.25	0.80
PVC	7.73	4.94	1.41

**Table 4:** Kinetic parameters for the pseudo first-order, pseudo second-order, and ID models.

Plastic Type	q_e_ (mg/kg)	Pseudo First-Order		Pseudo Second-Order		Intraparticle Diffusion (ID)	
		k (1/h)	R^2^	k (kg/mg h)	R^2^	k (mg/ kg h)	R^2^
PS	261.42	0.02	0.95	0.0038	0.99	12.00	0.64
PP	308.73	0.02	0.88	0.0032	0.99	15.00	0.67
LDPE	315.52	0.02	0.91	0.0031	0.99	9.00	0.69

**Table 5:** Concentration of organic compounds in real microplastic samples from four Mexican beaches.

Beach	Tamiahua	Tuxpan	La Barra de Sontecomapan	Coatzacoalcos
*q_e_* * (mg/kg)	35,258.81	16,133.76	1660.85	8776.99
Standard deviation (mg/kg)	12,921.23	2980.46	1114.73	3088.20

* Units of *q_e_* and standard deviation are mg of organic compound per kg of microplastic.

## Data Availability

Not applicable.
